# Supplementary material for: Prostate cancer reshapes the secreted and extracellular vesicle urinary proteomes
Source: Nat Commun. 2024 Jun 13;15:5069. doi: 10.1038/s41467-024-49424-5 (PMC11176296; doi:10.1038/s41467-024-49424-5)
Supplement: Supplementary file 1 — Supplementary Information [file 41467_2024_49424_MOESM1_ESM.pdf]

SUPPLEMENTARY INFORMATION:

Prostate Cancer Reshapes the Secreted and Extracellular Vesicle Urinary Proteomes

Amanda Khoo[1,2,+], Meinusha Govindarajan[1,2,+], Zhuyu Qiu[3,4,5,6,+], Lydia Y. Liu[1,2,3,4,5,6], Vladimir Ignatchenko[2], Matthew Waas[2], Andrew Macklin[2], Alexander Keszei[2], Sarah Neu[7], Brian P. Main[8], Lifang Yang[8,9], Raymond S. Lance[10], Michelle R. Downes[11,12], O. John Semmes[8,9], Danny Vesprini[12,13], Stanley K. Liu[1,13,14], Julius O. Nyalwidhe[8,9], Paul C. Boutros[1,3,4,5,6,15,16,*], Thomas Kislinger[1,2,*]

1 Department of Medical Biophysics, University of Toronto, Toronto, Ontario, M5G 1L7, Canada

2 Princess Margaret Cancer Centre, University Health Network, Toronto, Ontario, M5G 2C1, Canada

3 Jonsson Comprehensive Cancer Center, David Geffen School of Medicine, University of California, Los Angeles, Los Angeles, California 90024, USA.

4 Department of Human Genetics, University of California, Los Angeles, Los Angeles, California 90095, USA.

5 Department of Urology, University of California, Los Angeles, Los Angeles, California 90095, USA.

6 Institute for Precision Health, David Geffen School of Medicine, University of California, Los Angeles, Los Angeles, California 90095, USA.

7 Division of Surgery, Urology, Sunnybrook Health Sciences Centre, Toronto, ON M4N 3M5, Canada.

8 Leroy T. Canoles Jr. Cancer Research Center, Eastern Virginia Medical School, Norfolk, Virginia 23507, USA.

9 Department of Microbiology and Molecular Cell Biology, Eastern Virginia Medical School, Norfolk, Virginia 23507, USA.

10 Spokane Urology, Spokane, Washington 99202, USA.

11 Division of Anatomic Pathology, Laboratory Medicine and Molecular Diagnostics, Sunnybrook Health Sciences Centre, Toronto, ON M4N 3M5, Canada

12 Laboratory Medicine and Pathobiology, University of Toronto, Toronto, ON M5S 1A8, Canada.

13 Department of Radiation Oncology, University of Toronto, Toronto, Ontario, M5T 1P5, Canada.

14 Odette Cancer Research Program, Sunnybrook Research Institute, Toronto, Ontario, M4N 3M5, Canada.

[15] Department of Pharmacology and Toxicology, University of Toronto, Toronto, Ontario, M5S 1A8, Canada.

[16] Broad Stem Cell Research Center, University of California, Los Angeles, California, 90095, USA.

[+] These authors contributed equally

*Correspondence PBoutros@mednet.ucla.edu (P.C.B.), Thomas.kislinger@utoronto.ca (T.K.)

Supplementary Figures

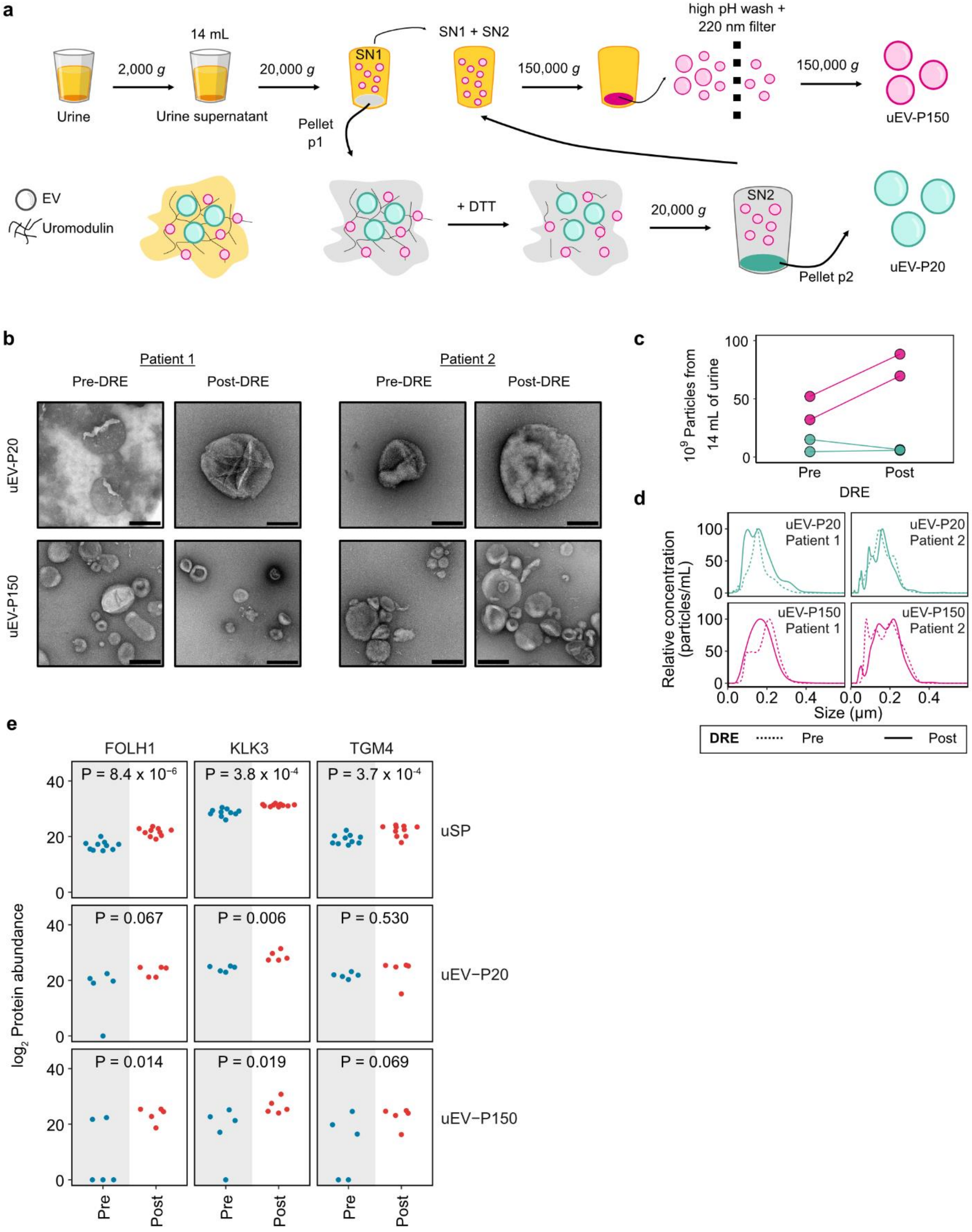
a
Urine
2,000 g
14 mL
Urine supernatant
20,000 g
SN1
Pellet p1
SN1 + SN2
150,000 g
high pH wash + 220 nm filter
150,000 g
uEV-P150
EV
Uromodulin
+ DTT
20,000 g
SN2
Pellet p2
uEV-P20
b
Patient 1
Pre-DRE
Post-DRE
Patient 2
Pre-DRE
Post-DRE
uEV-P20
uEV-P150
c
10^9 Particles from 14 mL of urine
Pre
Post
DRE
d
uEV-P20 Patient 1
uEV-P20 Patient 2
uEV-P150 Patient 1
uEV-P150 Patient 2
Relative concentration (particles/mL)
Size (μm)
DRE
Pre
Post
e
FOLH1
KLK3
TGM4
P = 8.4 x 10^-6
P = 3.8 x 10^-4
P = 3.7 x 10^-4
uSP
P = 0.067
P = 0.006
P = 0.530
uEV−P20
P = 0.014
P = 0.019
P = 0.069
uEV−P150
log2 Protein abundance
Pre
Post

Supplementary Fig. 1 **Molecular and biophysical characterization of uEVs. a** Urinary extracellular vesicle (uEV) isolation by differential ultracentrifugation and filtration. SN: supernatant; DTT: dithiothreitol. **b** Negative stain transmission electron microscopy images of uEVs isolated from pre- and post-DRE urine from two patients. Scale bar: 200 nm. **c** Number of uEVs isolated from 14 mL of urine from two men with cISUP Grade Group 1 tumors. **d** Size distribution of uEV-P20 and uEV-P150 from two matched patients by nanoparticle tracking analysis. **e** Protein abundance of select prostate proteins in pre- vs. post-DRE urine. P-values from a two-sided Wilcoxon signed-rank test. Source data are provided as a Source Data file.

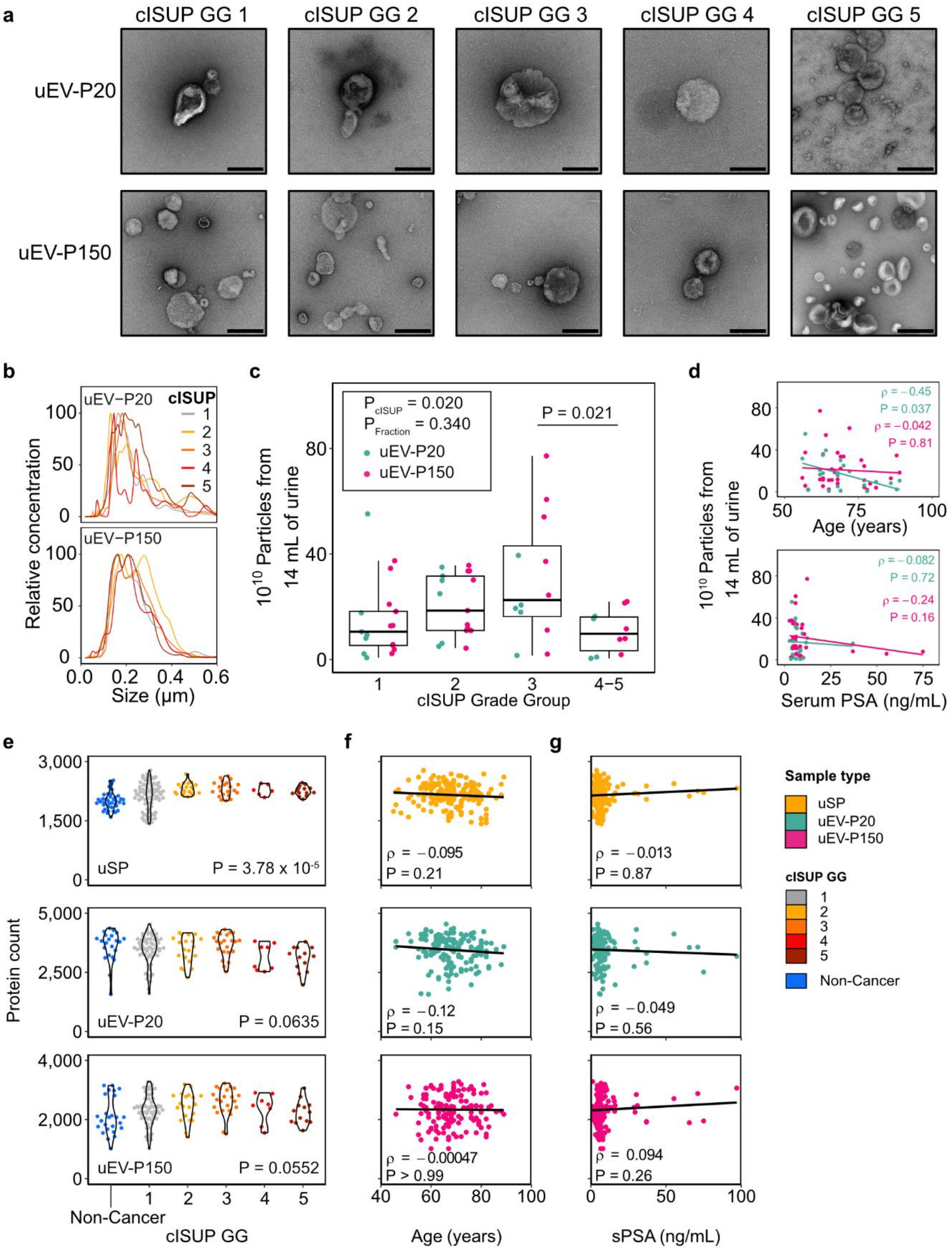

a
cISUP GG 1
cISUP GG 2
cISUP GG 3
cISUP GG 4
cISUP GG 5
uEV-P20
uEV-P150
b
uEV−P20
cISUP
1
2
3
4
5
uEV−P150
Relative concentration
Size (μm)
c
P_{cISUP} = 0.020
$P_{Fraction}$ = 0.340
uEV-P20
uEV-P150
P = 0.021
10^{10} Particles from 14 mL of urine
cISUP Grade Group
d
ρ = −0.45
P = 0.037
ρ = −0.042
P = 0.81
Age (years)
ρ = −0.082
P = 0.72
ρ = −0.24
P = 0.16
Serum PSA (ng/mL)
e
uSP
P = 3.78 x 10^{-5}
uEV-P20
P = 0.0635
uEV-P150
P = 0.0552
Protein count
Non-Cancer
cISUP GG
f
ρ = −0.095
P = 0.21
ρ = −0.12
P = 0.15
ρ = −0.00047
P > 0.99
Age (years)
g
ρ = −0.013
P = 0.87
ρ = −0.049
P = 0.56
ρ = 0.094
P = 0.26
sPSA (ng/mL)
Sample type
uSP
uEV-P20
uEV-P150
cISUP GG
1
2
3
4
5
Non-Cancer

Supplementary Fig. 2 **uEV associations with clinical covariates. a** Negative stain transmission electron microscopy (TEM) images of uEVs isolated from the post-DRE urine of men with different cISUP Grade Group (GG) tumors. Scale bar: 200 nm. n = 1 patient per cISUP GG. **b** Size distribution by NTA. Each curve represents the mean size for each cISUP GG. n_{GG1} = 32 patients, n_{GG2} = 21, n_{GG1} = 12, n_{GG4} = 4, n_{GG5} = 8. **c** Number of uEV-P20 and uEV-P150 isolated from 14 mL of post-DRE urine from men with different cISUP Grade Group tumors. Patients: uEV-$P20_{GG1}$ = 7, uEV-$P150_{GG1}$ = 10, uEV-$P20_{GG2}$ = 6, uEV-$P20_{GG2}$ = 11, uEV-$P20_{GG3}$ = 5, uEV-$P150_{GG3}$ = 7, uEV-$P20_{GG4-5}$ = 4, uEV-$P150_{GG4-5}$ = 6. Significantly different groups (two-way ANOVA with *post hoc* Tukey's HSD < 0.05) are labeled. **d** Number of uEV particles and age at diagnosis (top) or serum PSA level (bottom). **e-g** Associations between protein counts and cISUP GG (**e**), age (**f**) and serum PSA (**g**). P-values in (**e**) from one-way ANOVA. Spearman's ρ for (**f-g**). Patients: GG1 = 54, GG2 = 20, GG3 = 26, GG4 = 11, GG5 = 15. Source data are provided as a Source Data file.

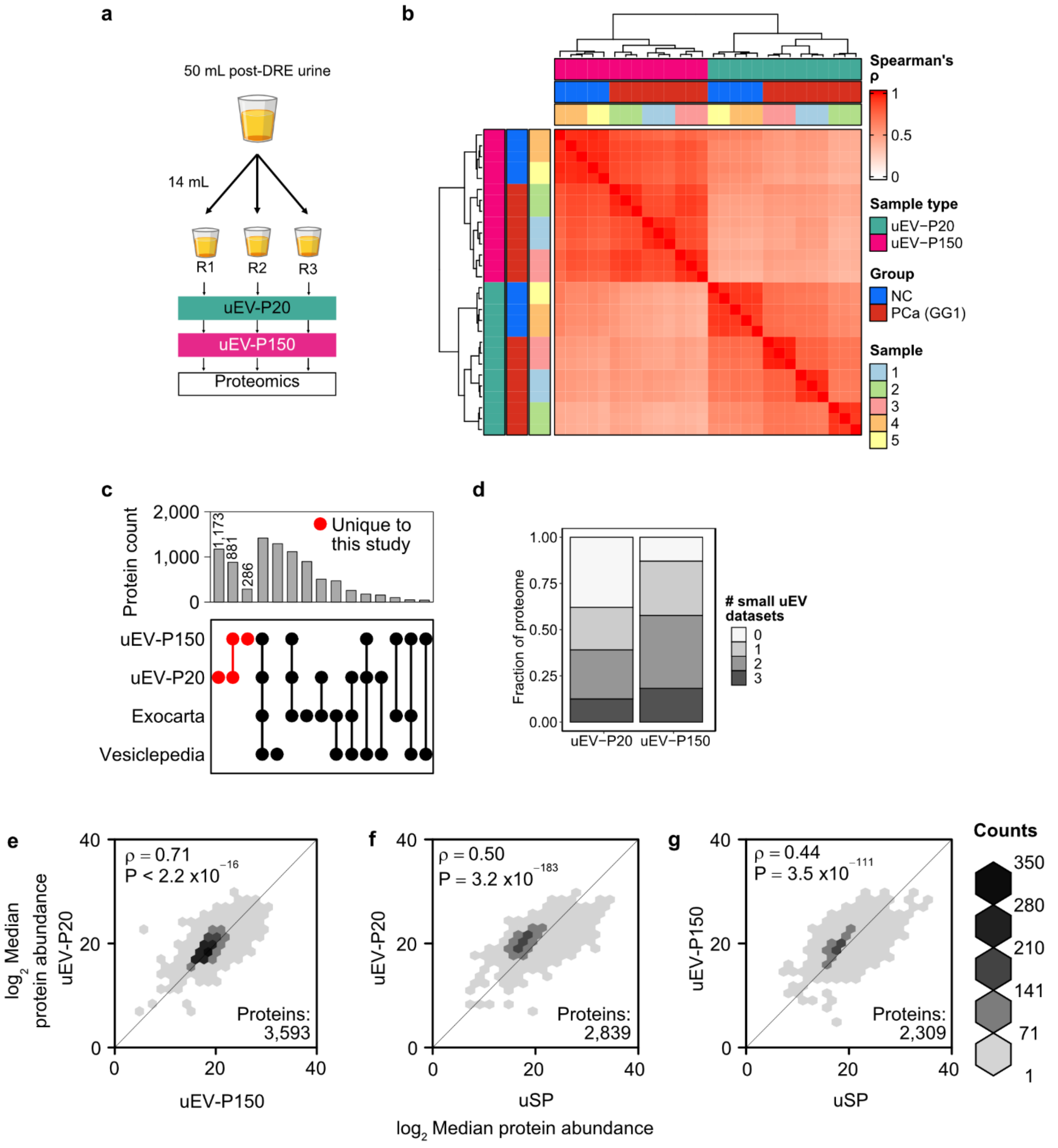
a
50 mL post-DRE urine
14 mL
R1
R2
R3
uEV-P20
uEV-P150
Proteomics
b
Spearman's ρ
1
0.5
0
Sample type
uEV−P20
uEV−P150
Group
NC
PCa (GG1)
Sample
1
2
3
4
5
c
Protein count
2,000
1,000
0
1,173
881
286
Unique to this study
uEV-P150
uEV-P20
Exocarta
Vesiclepedia
d
Fraction of proteome
1.00
0.75
0.50
0.25
0.00
uEV−P20
uEV−P150
small uEV datasets
0
1
2
3
e
ρ = 0.71
P < 2.2 x10^−16
Proteins: 3,593
log2 Median protein abundance uEV-P20
uEV-P150
f
ρ = 0.50
P = 3.2 x10^−183
Proteins: 2,839
uEV-P20
uSP
g
ρ = 0.44
P = 3.5 x10^−111
Proteins: 2,309
uEV-P150
uSP
log2 Median protein abundance
Counts
350
280
210
141
71
1

Supplementary Fig. 3 **uEV proteomes are distinct from uSP proteomes. a** Experimental design for assessing reproducibility of uEV isolation from post-DRE urine. **b** Pairwise comparisons (Spearman's ρ) between experimental replicates, clinical groups, and uEV fractions. NC: Non-cancer (two pooled samples); PCa: Prostate cancer (three cISUP Grade Group 1 patients). **c** Overlap in uEV proteins detected in this study with ExoCarta[1] and Vesiclepedia[2]. Proteins unique to the current study are in red. **d** Fraction of uEV proteins detected in this dataset that were also detected in three other published post-DRE urine small uEV datasets[3–5]. **e-g** Median protein abundance showing sample type correlation (Spearman's ρ) between **e** uEV-P20 vs. uEV-P150, **f** uEV-P20 vs. uSP and **g** uEV-P150 vs. uSP. *n* = 96 patients. Source data are provided as a Source Data file.

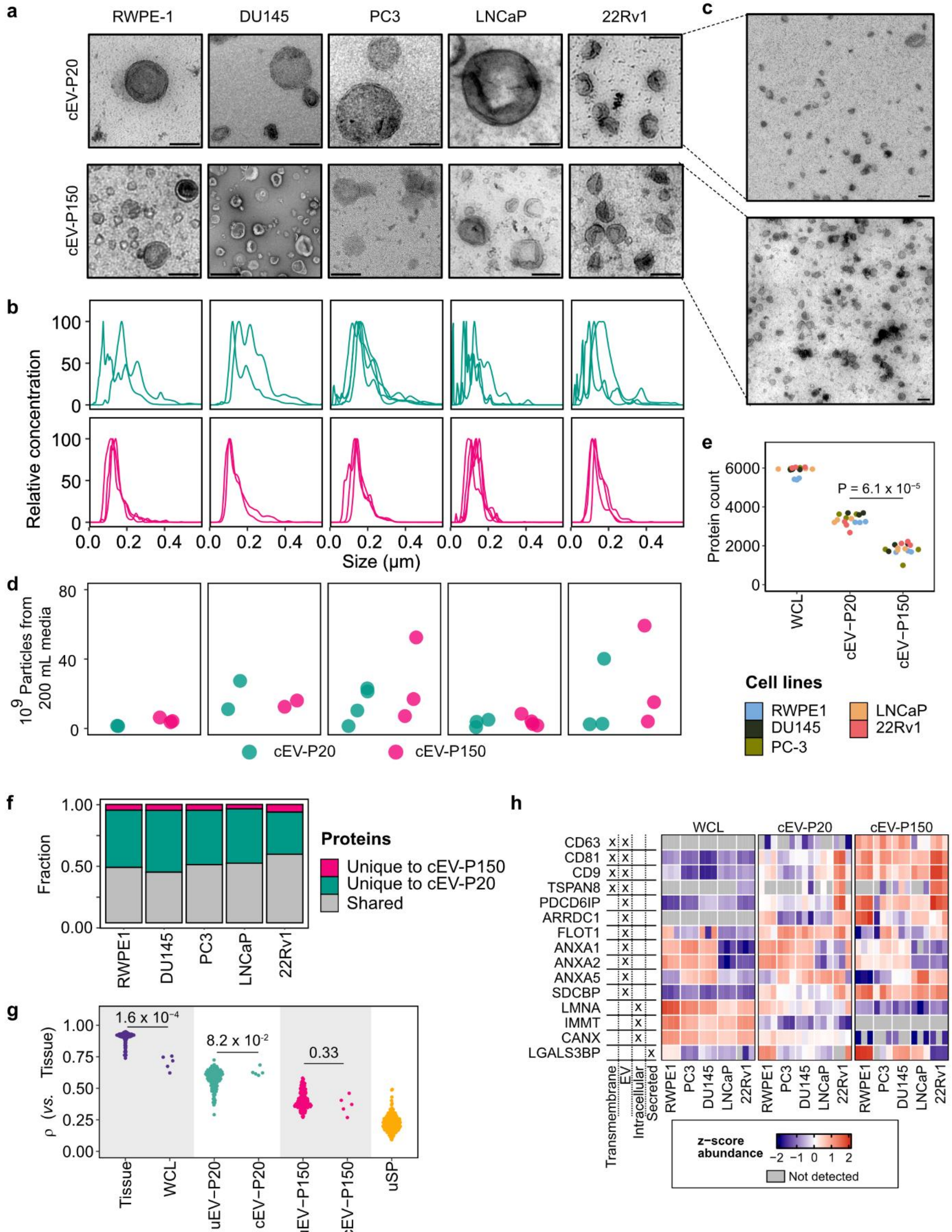

a
RWPE-1
DU145
PC3
LNCaP
22Rv1
cEV-P20
cEV-P150
c
b
Relative concentration
100
50
0
0.0
0.2
0.4
Size (μm)
d
10^9 Particles from 200 mL media
80
40
0
cEV-P20
cEV-P150
e
Protein count
6000
4000
2000
0
P = 6.1 x 10^{-5}
WCL
cEV−P20
cEV−P150
Cell lines
RWPE1
DU145
PC-3
LNCaP
22Rv1
f
Fraction
1.00
0.50
0.00
Proteins
Unique to cEV-P150
Unique to cEV-P20
Shared
RWPE1
DU145
PC3
LNCaP
22Rv1
g
ρ (vs. Tissue)
1.6 x 10^{-4}
8.2 x 10^{-2}
0.33
1.00
0.75
0.50
0.25
0.00
Tissue
WCL
uEV−P20
cEV−P20
uEV−P150
cEV−P150
uSP
h
WCL
cEV-P20
cEV-P150
CD63
CD81
CD9
TSPAN8
PDCD6IP
ARRDC1
FLOT1
ANXA1
ANXA2
ANXA5
SDCBP
LMNA
IMMT
CANX
LGALS3BP
Transmembrane
EV
Intracellular
Secreted
RWPE1
PC3
DU145
LNCaP
22Rv1
z−score abundance
−2 −1 0 1 2
Not detected

Supplementary Fig. 4 **Biophysics and proteomics of cell line-derived EVs. a** Negative stain TEM images of cell line EVs (cEV) isolated from conditioned media of prostate normal epithelium cell line (RWPE-1) and prostate cancer cell lines (DU145, PC3, LNCaP and 22Rv1). Scale bar: 200 nm. n = 1 experimental replicate per cell line. **b** Size distribution determined by nanoparticle tracking analysis. Each curve represents the mean size for each biological replicate of cEV-P20 (top) or cEV-P150 (bottom). Experimental replicates: cEV-$P20_{RWPE1}$ = 2, cEV-$P150_{RWPE1}$ = 3; cEV-$P20_{DU145}$ = 2, cEV-$P150_{DU145}$ = 2; cEV-$P20_{PC3}$ = 4, cEV-$P150_{PC3}$ = 3; cEV-$P20_{LNCaP}$ = 3, cEV-$P150_{LNCaP}$ = 4; cEV-$P20_{22Rv1}$ = 3, cEV-$P150_{22Rv1}$ = 3. **c** Wide-view negative stain TEM images of cEV-P20 (top) and cEV-P150 (bottom) isolated from conditioned media of 22Rv1 prostate cancer cell line. Scale bar: 200 nm. n = 1 experimental replicate per cell line. **d** Number of cEV-P20 and cEV-P150 particles determined by nanoparticle tracking analysis. Experimental replicates: cEV-$P20_{RWPE1}$ = 2, cEV-$P150_{RWPE1}$ = 3; cEV-$P20_{DU145}$ = 2, cEV-$P150_{DU145}$ = 2; cEV-$P20_{PC3}$ = 4, cEV-$P150_{PC3}$ = 3; cEV-$P20_{LNCaP}$ = 3, cEV-$P150_{LNCaP}$ = 4; cEV-$P20_{22Rv1}$ = 3, cEV-$P150_{22Rv1}$ = 3. **e** Protein counts for each cell line-derived sample type, colored by cell line. n = 3 experimental replicates per cell line. P-values from two-sided Wilcoxon signed-rank test. **f** Fraction of shared or unique proteins between cEV-P20 and cEV-P150 fractions across cell lines. **g** Spearman's ρ between $\log_2$ mean protein abundance in prostate tissue (157 samples), and individual samples from tissue, whole cell lysate (WCL, 5 cell lines), uEVs ($n_{uEV\text{-}P20}$ = 146 samples, $n_{cEV\text{-}P20}$ = 5 cell lines, $n_{uEV\text{-}P150}$ = 148, $n_{cEV\text{-}P150}$ = 5 cell lines), and soluble proteins (uSP, n = 175). Each data point from cell line samples represents the mean of 3 experimental replicates per cell line. P-values from two-sided Mann-Whitney U test. **h** z-scores of $\log_2$ protein abundance of select EV and non-EV markers (MISEV 2018[5]) in WCL, cEV-P20 and cEV-P150. Source data are provided as a Source Data file.

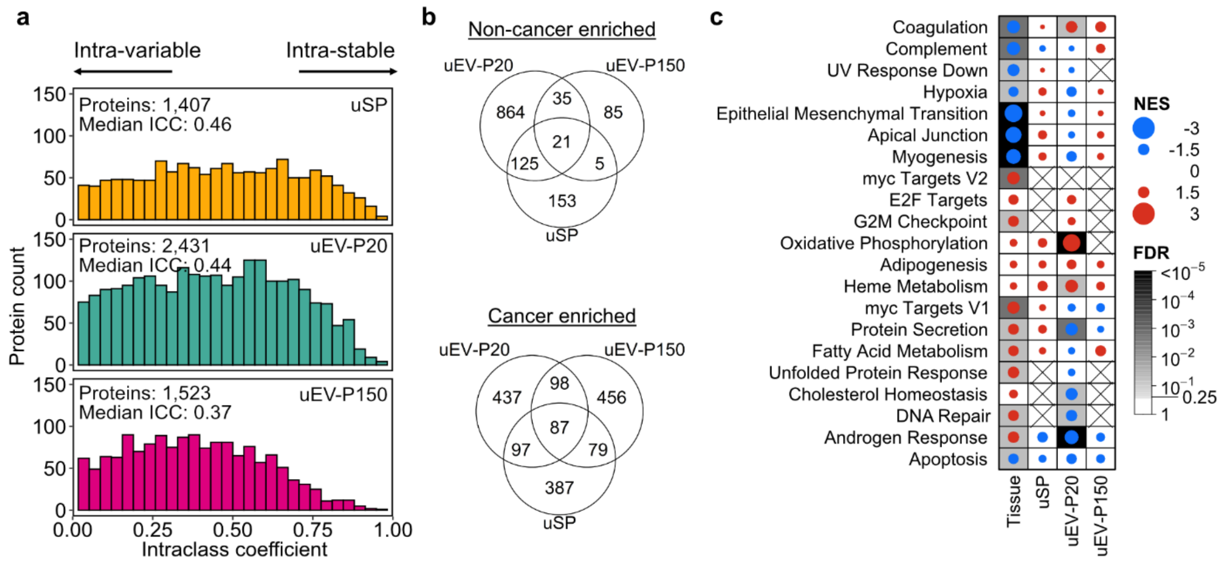

Supplementary Fig. 5 **Prostate cancer vs. non-cancer proteomes across sample types. a** Distribution of estimated variability for proteins in each urine fraction, estimated using intraclass correlation coefficient (ICC). *n = 5 patients.* **b** Overlap of proteins significantly upregulated in non-cancer fractions (top panel) and prostate cancer fractions (bottom panel) using a two-sided Mann-Whitney U test with Benjamini-Hochberg correction, FDR < 0.05. **c** Gene set enrichment analysis Hallmark-enriched gene sets in prostate cancers and non-cancers across sample types. Only gene sets that were significantly enriched (FDR < 0.25) in at least one sample type are shown. Crosses denote non-significant gene sets (FDR ≥ 0.25). uSP: urinary soluble proteins; uEV: urinary extracellular vesicles; NES: normalized enrichment score. Source data are provided as a Source Data file.

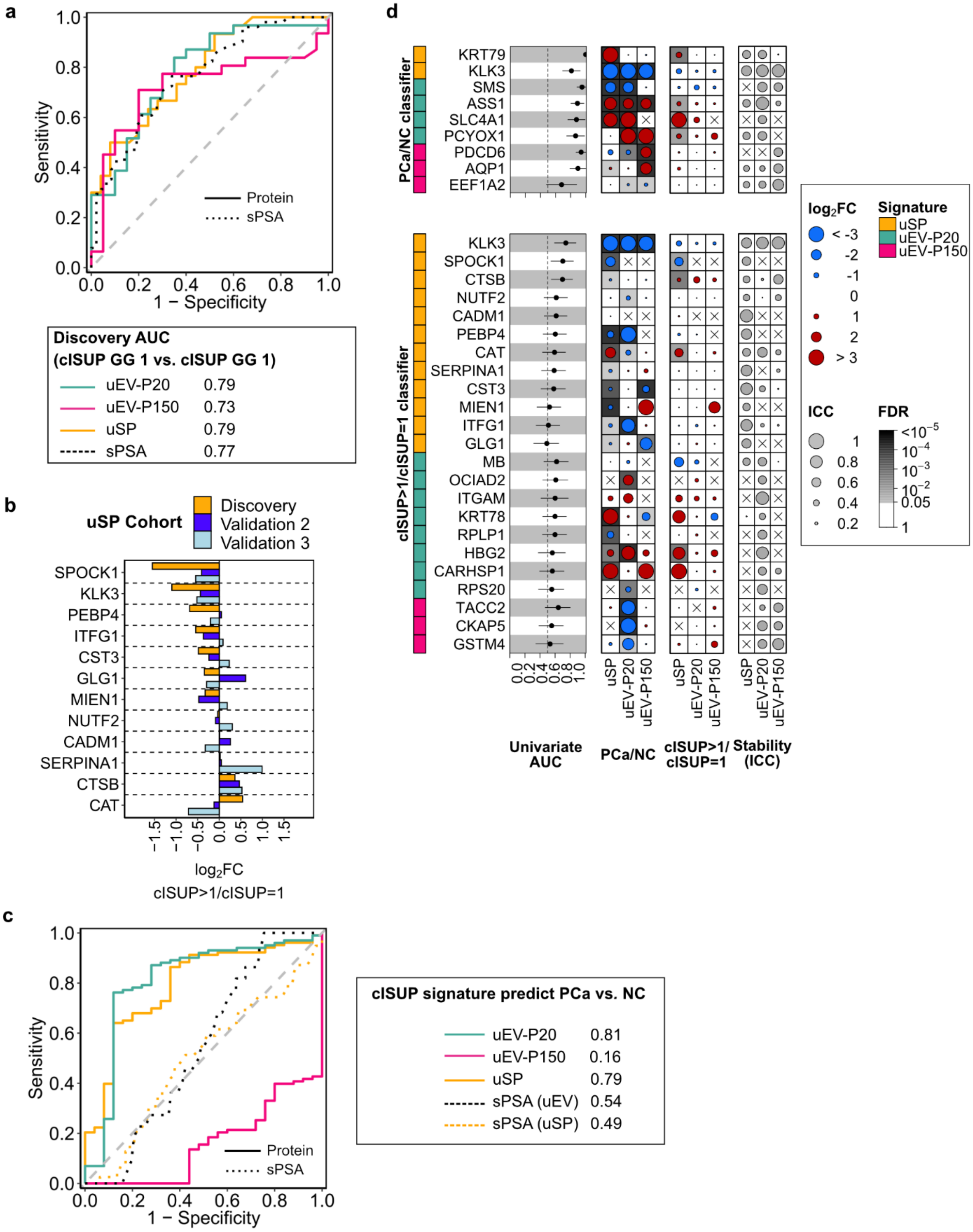
a
1.0
0.8
0.6
0.4
0.2
0.0
Sensitivity
0.0 0.2 0.4 0.6 0.8 1.0
1 − Specificity
Protein
sPSA
Discovery AUC
(cISUP GG 1 vs. cISUP GG 1)
uEV-P20 0.79
uEV-P150 0.73
uSP 0.79
sPSA 0.77
b
uSP Cohort
Discovery
Validation 2
Validation 3
SPOCK1
KLK3
PEBP4
ITFG1
CST3
GLG1
MIEN1
NUTF2
CADM1
SERPINA1
CTSB
CAT
−1.5 −1.0 −0.5 0.0 0.5 1.0 1.5
log2FC
cISUP>1/cISUP=1
c
Sensitivity
1 − Specificity
Protein
sPSA
cISUP signature predict PCa vs. NC
uEV-P20 0.81
uEV-P150 0.16
uSP 0.79
sPSA (uEV) 0.54
sPSA (uSP) 0.49
d
PCa/NC classifier
KRT79
KLK3
SMS
ASS1
SLC4A1
PCYOX1
PDCD6
AQP1
EEF1A2
cISUP>1/cISUP=1 classifier
KLK3
SPOCK1
CTSB
NUTF2
CADM1
PEBP4
CAT
SERPINA1
CST3
MIEN1
ITFG1
GLG1
MB
OCIAD2
ITGAM
KRT78
RPLP1
HBG2
CARHSP1
RPS20
TACC2
CKAP5
GSTM4
0.0 0.2 0.4 0.6 0.8 1.0
uSP uEV-P20 uEV-P150
Univariate AUC
PCa/NC
cISUP>1/ cISUP=1
Stability (ICC)
log2FC
< -3
-2
-1
0
1
2
> 3
Signature
uSP
uEV-P20
uEV-P150
ICC
1
0.8
0.6
0.4
0.2
FDR
<10−5
10−4
10−3
10−2
0.05
1

Supplementary Fig. 6 **Performance for cISUP Grade Group classifier a** Receiver operator characteristic (ROC) curve of multi-protein model performance in classifying cISUP GG>1 or cISUP GG 1. Dotted lines show performance of serum PSA. **b** log_2 fold changes of proteins in the uSP multi-protein model (**Supplementary Fig. 6a**) in the discovery cohort and two independent validation cohorts. Patients in Discovery: cISUP GG > 1 = 61, cISUP GG 1 = 50; Validation 1: cISUP GG > 1 = 114, cISUP GG 1 = 85; Validation 2: cISUP GG > 1 = 42, cISUP GG 1 = 28. **c** The performance (AUC) for all signature proteins in a univariate model for classifying patients with cISUP GG > 1 or GG 1 tumors with differences in protein abundance and intraclass coefficient (ICC). Grey background in dotmap denotes FDR < 0.05. Crosses denote proteins not detected in half of samples in that urine fraction. **d** ROC curve of cISUP GG protein model performance in the discovery cohort for classifying prostate cancers (PCa) vs. non-cancers (NC). Dotted lines show performance of serum PSA for uEVs (patients with matched uEV-P20 and uEV-P150) and uSP. Source data are provided as a Source Data file.

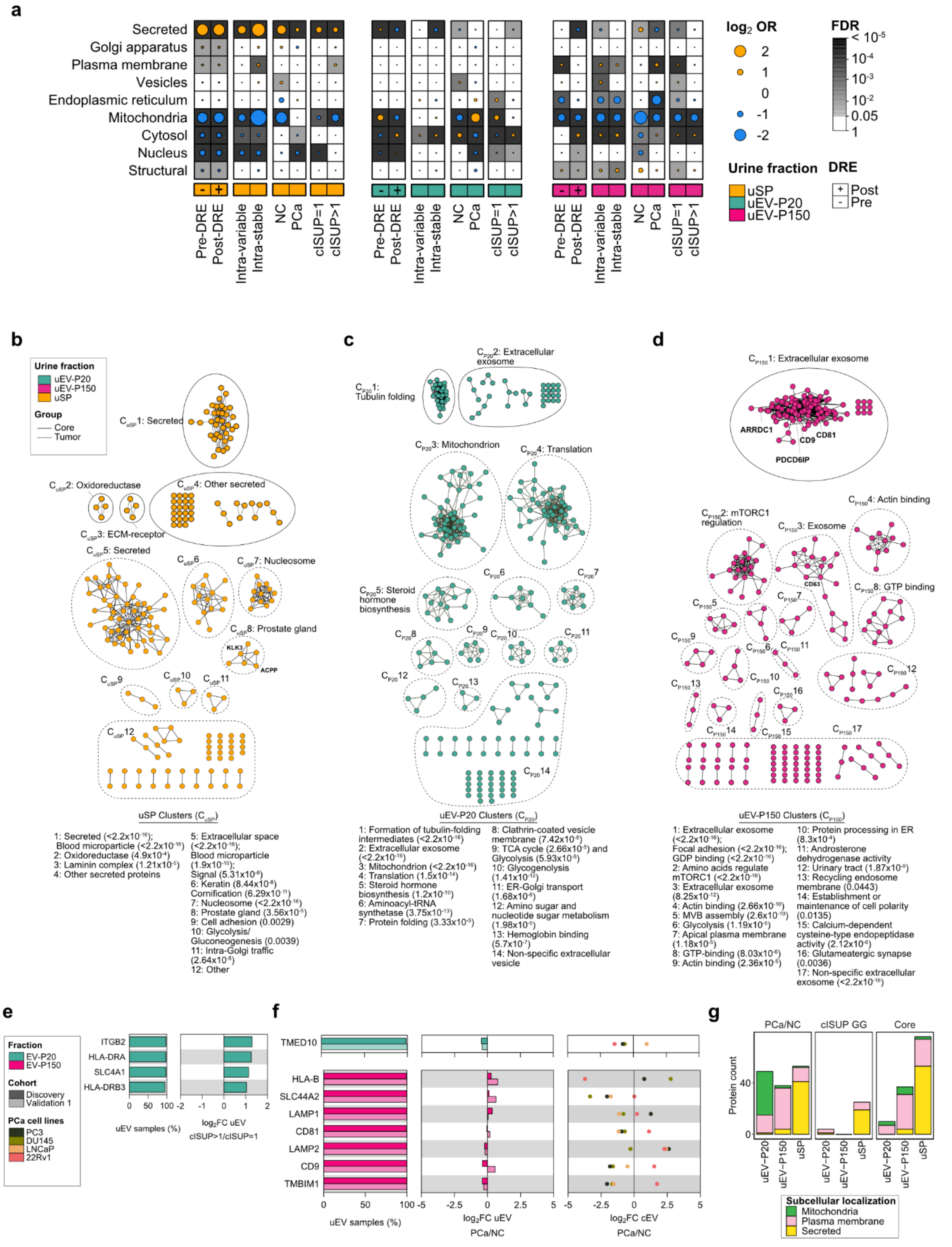
a
Secreted
Golgi apparatus
Plasma membrane
Vesicles
Endoplasmic reticulum
Mitochondria
Cytosol
Nucleus
Structural
Pre-DRE
Post-DRE
Intra-variable
Intra-stable
NC
PCa
cISUP=1
cISUP>1
log2 OR
2
1
0
-1
-2
FDR
< 10-5
10-4
10-3
10-2
0.05
1
Urine fraction
uSP
uEV-P20
uEV-P150
DRE
+ Post
- Pre
b
Urine fraction
uEV-P20
uEV-P150
uSP
Group
Core
Tumor
CuSP1: Secreted
CuSP2: Oxidoreductase
CuSP3: ECM-receptor
CuSP4: Other secreted
CuSP5: Secreted
CuSP6
CuSP7: Nucleosome
CuSP8: Prostate gland
KLK3
ACPP
CuSP9
CuSP10
CuSP11
CuSP12
uSP Clusters (CuSP)
1: Secreted (<2.2x10-16);
Blood microparticle (<2.2x10-16)
2: Oxidoreductase (4.9x10-4)
3: Laminin complex (1.21x10-5)
4: Other secreted proteins
5: Extracellular space
(<2.2x10-16);
Blood microparticle
(1.9x10-10);
Signal (5.31x10-8)
6: Keratin (8.44x10-8)
Cornification (6.29x10-11)
7: Nucleosome (<2.2x10-16)
8: Prostate gland (3.56x10-5)
9: Cell adhesion (0.0029)
10: Glycolysis/
Gluconeogenesis (0.0039)
11: Intra-Golgi traffic
(2.64x10-5)
12: Other
c
CP201: Tubulin folding
CP202: Extracellular exosome
CP203: Mitochondrion
CP204: Translation
CP205: Steroid hormone biosynthesis
CP206
CP207
CP208
CP209
CP2010
CP2011
CP2012
CP2013
CP2014
uEV-P20 Clusters (CP20)
1: Formation of tubulin-folding
intermediates (<2.2x10-16)
2: Extracellular exosome
(<2.2x10-16)
3: Mitochondrion (<2.2x10-16)
4: Translation (1.5x10-14)
5: Steroid hormone
biosynthesis (1.2x10-10)
6: Aminoacyl-tRNA
synthetase (3.75x10-13)
7: Protein folding (3.33x10-5)
8: Clathrin-coated vesicle
membrane (7.42x10-8)
9: TCA cycle (2.66x10-5) and
Glycolysis (5.93x10-5)
10: Glycogenolysis
(1.41x10-12)
11: ER-Golgi transport
(1.68x10-6)
12: Amino sugar and
nucleotide sugar metabolism
(1.98x10-5)
13: Hemoglobin binding
(5.7x10-7)
14: Non-specific extracellular
vesicle
d
CP1501: Extracellular exosome
ARRDC1
CD9
CD81
PDCD6IP
CP1502: mTORC1 regulation
CP1503: Exosome
CD63
CP1504: Actin binding
CP1505
CP1507
CP1508: GTP binding
CP1509
CP1506
CP15011
CP15010
CP15012
CP15013
CP15016
CP15014
CP15015
CP15017
uEV-P150 Clusters (CP150)
1: Extracellular exosome
(<2.2x10-16);
Focal adhesion (<2.2x10-16);
GDP binding (<2.2x10-16)
2: Amino acids regulate
mTORC1 (<2.2x10-16)
3: Extracellular exosome
(8.25x10-12)
4: Actin binding (2.66x10-10)
5: MVB assembly (2.6x10-10)
6: Glycolysis (1.19x10-5)
7: Apical plasma membrane
(1.18x10-5)
8: GTP-binding (8.03x10-6)
9: Actin binding (2.36x10-5)
10: Protein processing in ER
(8.3x10-4)
11: Androsterone
dehydrogenase activity
12: Urinary tract (1.87x10-8)
13: Recycling endosome
membrane (0.0443)
14: Establishment or
maintenance of cell polarity
(0.0135)
15: Calcium-dependent
cysteine-type endopeptidase
activity (2.12x10-6)
16: Glutameatergic synapse
(0.0036)
17: Non-specific extracellular
exosome (<2.2x10-16)
e
Fraction
EV-P20
EV-P150
Cohort
Discovery
Validation 1
PCa cell lines
PC3
DU145
LNCaP
22Rv1
ITGB2
HLA-DRA
SLC4A1
HLA-DRB3
uEV samples (%)
log2FC uEV
cISUP>1/cISUP=1
f
TMED10
HLA-B
SLC44A2
LAMP1
CD81
LAMP2
CD9
TMBIM1
uEV samples (%)
log2FC uEV
PCa/NC
log2FC cEV
PCa/NC
g
PCa/NC
cISUP GG
Core
Protein count
uEV-P20
uEV-P150
uSP
Subcellular localization
Mitochondria
Plasma membrane
Secreted

Supplementary Fig. 7 **Pathways implicated in EV cargo dysregulation. a** Dot plot of $\log_2$ odds ratio (OR) of over-representation for each term. Background shading indicates significant terms (FDR < 0.05, Fisher's exact test). **b-d** STRING and pathway analysis of proteins in uSP (**b**), uEV-P20 (**c**) or uEV-P150 (**d**) belonging to core or tumor gene sets (i.e., differentially abundant in PCa/NC). Nodes represent proteins, and edges represent protein-protein associations[6]. Each cluster is annotated with pathway terms and P-value in parentheses. **e** Summary of uEV-P20 grade markers with predicted cell surface localization from **Figure 7c**. Proteins are annotated with the frequency of detection in each fraction (left panel), and differential abundance in cISUP GG > 1 vs. GG 1 (right panel). **f** Summary of core uEV proteins with predicted cell surface localization from **Figure 7c**, annotated with the frequency of detection in each fraction (left panel), differential abundance in PCa vs. NC uEVs, cISUP GG uEVs, and PCa vs. NC cEVs. uEV: urinary EV, cEV: cell line EV. **g** Subcellular annotation[7] of all disease-specific and core markers for all fractions. Source data are provided as a Source Data file.

Supplementary References

1. Keerthikumar, S. *et al.* ExoCarta: A Web-Based Compendium of Exosomal Cargo. *J. Mol. Biol.* **428**, 688–692 (2016).
2. Kalra, H. *et al.* Vesiclepedia: a compendium for extracellular vesicles with continuous community annotation. *PLoS Biol.* **10**, e1001450 (2012).
3. Correll, V. L. *et al.* Optimization of small extracellular vesicle isolation from expressed prostatic secretions in urine for in-depth proteomic analysis. *J. Extracell. Vesicles* **11**, e12184 (2022).
4. Dhondt, B. *et al.* Unravelling the proteomic landscape of extracellular vesicles in prostate cancer by density-based fractionation of urine. *J. Extracell. Vesicles* **9**, 1736935 (2020).
5. Sinha, A. *et al.* The Proteogenomic Landscape of Curable Prostate Cancer. *Cancer Cell* **35**, 414-427.e6 (2019).
6. Szklarczyk, D. *et al.* The STRING database in 2023: protein-protein association networks and functional enrichment analyses for any sequenced genome of interest. *Nucleic Acids Res.* **51**, D638–D646 (2023).
7. Thul, P. J. *et al.* A subcellular map of the human proteome. *Science* **356**, eaal3321 (2017).
