# Supplementary material for: Prostate cancer reshapes the secreted and extracellular vesicle urinary proteomes
Source: Nat Commun. 2024 Jun 13;15:5069. doi: 10.1038/s41467-024-49424-5 (PMC11176296; doi:10.1038/s41467-024-49424-5)
Supplement: Supplementary file 3 — Description of Additional Supplementary Files [file 41467_2024_49424_MOESM3_ESM.pdf]

Description of Additional Supplementary Files

Supplementary Data 1. Patient characteristics.

Supplementary Data 2. Processed proteomic profiles of urines, uEVs and cEVs.

Supplementary Data 3. Results from pathway analysis.

Supplementary Data 4. Results from statistical analyses.

Supplementary Data 5. Mass spectrometry method details.
