# Supplementary material for: Prostate cancer reshapes the secreted and extracellular vesicle urinary proteomes
Source: Nat Commun. 2024 Jun 13;15:5069. doi: 10.1038/s41467-024-49424-5 (PMC11176296; doi:10.1038/s41467-024-49424-5)
Supplement: Supplementary file 9 — Reporting Summary [file 41467_2024_49424_MOESM9_ESM.pdf]

nature portfolio

Corresponding author(s): Paul C. Boutros, Thomas Kislinger

Last updated by author(s): Apr 30, 2024

Reporting Summary

Nature Portfolio wishes to improve the reproducibility of the work that we publish. This form provides structure for consistency and transparency in reporting. For further information on Nature Portfolio policies, see our Editorial Policies and the Editorial Policy Checklist.

Statistics

For all statistical analyses, confirm that the following items are present in the figure legend, table legend, main text, or Methods section.

n/a	Confirmed	
☐	☒	The exact sample size (n) for each experimental group/condition, given as a discrete number and unit of measurement
☐	☒	A statement on whether measurements were taken from distinct samples or whether the same sample was measured repeatedly
☐	☒	The statistical test(s) used AND whether they are one- or two-sided<br>*Only common tests should be described solely by name; describe more complex techniques in the Methods section.*
☐	☒	A description of all covariates tested
☐	☒	A description of any assumptions or corrections, such as tests of normality and adjustment for multiple comparisons
☐	☒	A full description of the statistical parameters including central tendency (e.g. means) or other basic estimates (e.g. regression coefficient) AND variation (e.g. standard deviation) or associated estimates of uncertainty (e.g. confidence intervals)
☐	☒	For null hypothesis testing, the test statistic (e.g. F, t, r) with confidence intervals, effect sizes, degrees of freedom and P value noted<br>*Give P values as exact values whenever suitable.*
☒	☐	For Bayesian analysis, information on the choice of priors and Markov chain Monte Carlo settings
☒	☐	For hierarchical and complex designs, identification of the appropriate level for tests and full reporting of outcomes
☐	☒	Estimates of effect sizes (e.g. Cohen's d, Pearson's r), indicating how they were calculated

Our web collection on statistics for biologists contains articles on many of the points above.

Software and code

Policy information about availability of computer code

Data collection	N/A
Data analysis	All relevant software and programs utilized include:<br>MaxQuant (v.1.5.8.3 and 1.6.2.3), R statistical environment (v.4.2.1), R packages BoutrosLab Plotting General package22 (v.7.0.3), ggplot2 (3.4.0), ggbeeswarm (v.0.6.0), ggpubr (v.0.4.0), ComplexHeatmap (v.2.12.1), gprofiler2 (v.0.2.1), caret (v.6.0.91), pROC (v.1.18.0) EnrichmentMap (v.3.3.4), Cytoscape (v.3.9.1),<br>Further details have been included in the materials and methods section.

For manuscripts utilizing custom algorithms or software that are central to the research but not yet described in published literature, software must be made available to editors and reviewers. We strongly encourage code deposition in a community repository (e.g. GitHub). See the Nature Portfolio guidelines for submitting code & software for further information.

Data

Policy information about availability of data

All manuscripts must include a data availability statement. This statement should provide the following information, where applicable:

- Accession codes, unique identifiers, or web links for publicly available datasets
- A description of any restrictions on data availability
- For clinical datasets or third party data, please ensure that the statement adheres to our policy

The mass spectrometry raw data generated in this study have been deposited in the UCSD MassIVE database under accession code MSV000092061 [ftp://massive.ucsd.edu/MSV000092061/]. The processed proteomics data are provided in Supplementary Data 2. All relevant data for the EV experiments have been submitted to the EV-TRACK knowledgebase61 (EV-TRACK ID: EV230578). An accompanying website for this study provides an interactive browser for interrogation of multiple datasets and clinical cohorts, available at http://kislingerlab.uhnres.utoronto.ca/ev/home/. Published proteomics datasets used in this study2,17,18,33 can be accessed at UCSD's MassIVE database under accession codes MSV000088000 [ftp://massive.ucsd.edu/MSV000088000/] and MSV000081552 [ftp://massive.ucsd.edu/MSV000081552/] or PRIDE repository PXD015289 [https://proteomecentral.proteomexchange.org/cgi/GetDataset?ID=PXD015289] and PXD026974 [https://proteomecentral.proteomexchange.org/cgi/GetDataset?ID=PXD026974]. Source data are provided with this study.

Research involving human participants, their data, or biological material

Policy information about studies with human participants or human data. See also policy information about sex, gender (identity/presentation), and sexual orientation and race, ethnicity and racism.

Reporting on sex and gender	No sex-based analysis was performed as all patients were male. No gender-based analysis was performed as information on gender was not recorded for this cohort.
Reporting on race, ethnicity, or other socially relevant groupings	No race or ethnicity-based analysis was done as this data was not available for this cohort.
Population characteristics	The population included treatment naive patients with localized prostate cancer at diagnosis.<br>- Patient ages 39-89 (median 65)<br>- Serum prostate specific antigen 0.5-97 (median 5.7)<br>- 78 patients had benign prostatic hyperplasia or had elevated serum PSA levels but had no detectable cancer on systematic needle biopsy. 426 patients had detectable prostate cancer on needle biopsy.<br>- Prostate cancer patients spanned all clinical ISUP Grade Groups 1-5, Clinical T category 1-4 and D'Amico Risk Group Low, Intermediate and High. No patients had nodal or metastatic disease at diagnosis.<br>- All other information on the patient cohort can be found in Methods and Supplementary Table 1.
Recruitment	This study included patients with diagnosed prostate cancer at Eastern Virginia Medical School, Norfolk, Virginia, USA and Sunnybrook Health Sciences Centre, Toronto, Ontario, Canada with informed consent and use of Institutional Review Board approved protocols at both institutes. Men with benign prostatic conditions included individuals with elevated serum PSA levels but no diagnosed prostate cancer on transrectal ultrasound-guided 12-core biopsy (Biopsy-negative; 20 patients; median sPSA 5.2 ng/mL, range 0.5 – 31.5 ng/mL), or benign prostatic hyperplasia (BPH; 58 patients; median sPSA 5.9 ng/mL, range 0.5 – 15.1 ng/mL). Selection criteria for men with benign prostatic conditions included a diagnostic sPSA level < 20 ng/mL and post-surgery sPSA level <0.1 ng/mL to exclude highly metastatic men.
Ethics oversight	Ethics protocols were approved by the ethics boards at Eastern Virginia Medical School, Norfolk, Virginia, USA and Sunnybrook Health Sciences Centre, Toronto, Ontario, Canada

Note that full information on the approval of the study protocol must also be provided in the manuscript.

Field-specific reporting

Please select the one below that is the best fit for your research. If you are not sure, read the appropriate sections before making your selection.

☒ Life sciences ☐ Behavioural & social sciences ☐ Ecological, evolutionary & environmental sciences

For a reference copy of the document with all sections, see nature.com/documents/nr-reporting-summary-flat.pdf

Life sciences study design

All studies must disclose on these points even when the disclosure is negative.

Sample size	Figure legends indicate sample sizes for each experiment. No statistical methods to estimate sample size were used. Instead, data from all cancer cases obtained from the cited repositories were analyzed and p-values from statistical tests used to assess statistical significance and appropriateness of sample sizes
Data exclusions	No data was excluded.

Replication	Reproducibility of EV isolation and proteomics was assessed in Supplementary Fig. 2a-b.<br>cell line EV proteomics were performed in triplicate.<br>Nanoparticle tracking analysis for uEVs and cEVs were performed with 22-34 patients (for uEV) and 2-4 experimental replicates (for cEV).<br>Transmission electron microscopy of uEVs and cEVs were performed with 1-3 replicates (Figures 1a-b, 2d, Supplementary Fig. 2a, 4a).
Randomization	Samples were randomized for sample preparation and mass spectrometry analysis.
Blinding	Analysts were blinded to groups at sample preparation and data acquisition.

Reporting for specific materials, systems and methods

We require information from authors about some types of materials, experimental systems and methods used in many studies. Here, indicate whether each material, system or method listed is relevant to your study. If you are not sure if a list item applies to your research, read the appropriate section before selecting a response.

Materials & experimental systems

n/a	Involved in the study
☒	☐ Antibodies
☐	☒ Eukaryotic cell lines
☒	☐ Palaeontology and archaeology
☒	☐ Animals and other organisms
☒	☐ Clinical data
☒	☐ Dual use research of concern
☒	☐ Plants

Methods

n/a	Involved in the study
☒	☐ ChIP-seq
☒	☐ Flow cytometry
☒	☐ MRI-based neuroimaging

Eukaryotic cell lines

Policy information about cell lines and Sex and Gender in Research

Cell line source(s)	Commercial human prostate cell lines DU145, (ATCC #HTB-81) PC3 (ATCC #CRL-1435), 22Rv1 (ATCC #CRL-2505), LNCaP (ATCC #CRL-1740), and RWPE-1 (ATCC #CRL-3607) were a gift from Dr. Stanley Liu, Sunnybrook Health Sciences Centre, Toronto, Ontario, Canada.
Authentication	Cell lines were authenticated using short tandem repeat analysis.
Mycoplasma contamination	Cell lines were regularly tested for mycoplasma prior to use in experiments and were free from contamination.
Commonly misidentified lines<br>(See ICLAC register)	No commonly misidentified cell lines were used.

Plants

Seed stocks	*Report on the source of all seed stocks or other plant material used. If applicable, state the seed stock centre and catalogue number. If plant specimens were collected from the field, describe the collection location, date and sampling procedures.*
Novel plant genotypes	*Describe the methods by which all novel plant genotypes were produced. This includes those generated by transgenic approaches, gene editing, chemical/radiation-based mutagenesis and hybridization. For transgenic lines, describe the transformation method, the number of independent lines analyzed and the generation upon which experiments were performed. For gene-edited lines, describe the editor used, the endogenous sequence targeted for editing, the targeting guide RNA sequence (if applicable) and how the editor was applied.*
Authentication	*Describe any authentication procedures for each seed stock used or novel genotype generated. Describe any experiments used to assess the effect of a mutation and, where applicable, how potential secondary effects (e.g. second site T-DNA insertions, mosiacism, off-target gene editing) were examined.*
